# Generation of Small ^32^P-Labeled Peptides as a Potential Approach to Colorectal Cancer Therapy

**DOI:** 10.1371/journal.pone.0002508

**Published:** 2008-06-25

**Authors:** John M. Abraham, Yulan Cheng, James P. Hamilton, Bogdan Paun, Zhe Jin, Rachana Agarwal, Takatsugu Kan, Stefan David, Alexandru Olaru, Jian Yang, Tetsuo Ito, Florin M. Selaru, Yuriko Mori, Stephen J. Meltzer

**Affiliations:** 1 Department of Medicine, The Johns Hopkins University School of Medicine, Baltimore, Maryland, United States of America; 2 Sidney Kimmel Comprehensive Cancer Center, The Johns Hopkins University School of Medicine, Baltimore, Maryland, United States of America; University of Arkansas, United States of America

## Abstract

Cancers have been revealed to be extremely heterogenous in terms of the frequency and types of mutations present in cells from different malignant tumors. Thus, it is likely that uniform clinical treatment is not optimal for all patients, and that the development of individualized therapeutic regimens may be beneficial. We describe the generation of multiple, unique small peptides nine to thirty-four amino acids in length which, when labeled with the radioisotope ^32^P, bind with vastly differing efficiencies to cell lines derived from different colon adenocarcinomas. In addition, the most effective of these peptides permanently transfers the ^32^P radioisotope to colorectal cancer cellular proteins within two hours at a rate that is more than 150 times higher than in cell lines derived from other cancers or from the normal tissues tested. Currently, the only two FDA-approved radioimmunotherapeutic agents in use both employ antibodies directed against the B cell marker CD20 for the treatment of non-Hodgkin's lymphoma. By using the method described herein, large numbers of different ^32^P-labeled peptides can be readily produced and assayed against a broad spectrum of cancer types. This report proposes the development and use of ^32^P-labeled peptides as potential individualized peptide-binding therapies for the treatment of colon adenocarcinoma patients.

## Introduction

Recent landmark discoveries have convincingly documented the extensive genetic heterogeneity among human cancers, particularly colorectal tumors, by establishing the existence of a small number of frequently mutated gene “mountains” and a much higher number of gene “hills” mutated at much lower frequencies [1], [2]. This high degree of diversity among human colorectal cancers suggests that individualized treatment strategies hold great promise in successful clinical intervention. Several anticancer immunotherapies are currently in use, including Herceptin, Rituxin, and Avastin, a monoclonal antibody directed against VEGF (vascular endothelial growth factor) that is approved for colorectal cancer treatment [3]–[9].

Radioimmunotherapy (RIT) is an emerging technology with thus far only two FDA-approved protocols, both directed against non-Hodgkin's lymphoma (NHL). Each protocol utilizes a monoclonal antibody directed against the CD20 B-cell marker and can deliver ^90^Y (Zevalin) or ^131^I (Bexxar), each of which generates electrons (beta particles) that damage DNA, resulting in cell death [10], [11]. Currently, no RIT has yet been approved for the treatment of colorectal cancer [12].

We recently reported a set of nine different decapeptides, each varying from the others by only one to three amino acids, which when labeled with the beta-emitter ^32^P, bound to and permanently delivered, to varying degrees, this radioisotope to cell lines derived from a panel of different colorectal adenocarcinomas [13]. The most efficient ^32^P-labeled decapeptide resulted in permanent incorporation of radioisotope into colon adenocarcinoma cellular proteins at a rate over 100 times greater than in cell lines derived from a variety of other cancers or from normal colon, kidney or esophagus.

Herein, we report a class of slightly larger peptides (up to 34 amino acids long) that contain widely disparate peptide sequences, resulting in a broad range of cellular binding and radioisotope uptake properties. Each 34 amino acid peptide contains a nine amino acid core at its amino end to enable ^32^P labeling by protein kinase A, an eight amino acid core at its carboxy end, and up to 17 additional amino acids that dramatically alter both its binding to cells and its permanent incorporation of radioisotope into colon cancer cellular proteins. These results support further exploration of this strategy to develop potential new individualized therapeutic regimens against colon cancers.

## Results

### Production of ^32^P-labeled peptides and binding to colon adenocarcinoma cells

**Figure 1:**
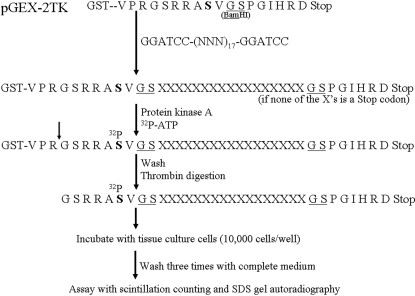
Schematic diagram of experimental approach. A PCR product containing 17 random codons was inserted into the BamHI site of pGEX-2TK producing various glutathione-S-transferase fusion proteins which were bound to glutathione-sepharose, and labeled with ^32^P using protein kinase A. After washing and thrombin digestion, the labeled peptides were incubated with several different cell lines and assayed.

Previously, we reported the discovery of nine different ^32^P-labeled decapeptides, each varying from one another by only one to three amino acids, that exhibited widely disparate abilities to bind to and transfer radioisotope permanently to proteins in cell lines established from a panel of colon adenocarcinomas. The most efficient of these ^32^P-labeled decapeptides permanently delivered radioisotope to colon cancer cells more than 100 times more efficiently than to cell lines derived from other cancers or the normal tissues tested. Herein, we report the production and identification of a new series of peptides, up to 34 amino acids in length, whose amino acid sequences dramatically alter their ability to bind to and permanently facilitate ^32^P incorporation into cells. **Figure 1** is a schematic representation of the experimental design, illustrating the cloning of a DNA fragment containing 17 randomly generated codons into the BamHI restriction enzyme site of pGEX-2TK. After bacterial transformation, individual clones were selected and expanded to produce a diverse set of ^32^P-labeled peptides. If no stop codons were present in the random DNA sequence, then a 34-residue peptide was generated, flanked at its amino end by the 9-residue protein kinase A labeling motif and at its carboxy terminus by an 8-residue sequence. As expected, in several clones, a stop codon was inserted, resulting in truncated peptides; however, all of these truncated peptides contained the protein kinase A substrate moiety. These diverse peptides were incubated with several different cell lines for two hours, adherent cells were washed three times, and radioactivity remaining bound to cells was assayed either immediately, or following overnight incubation in complete medium.

**Figure 2:**
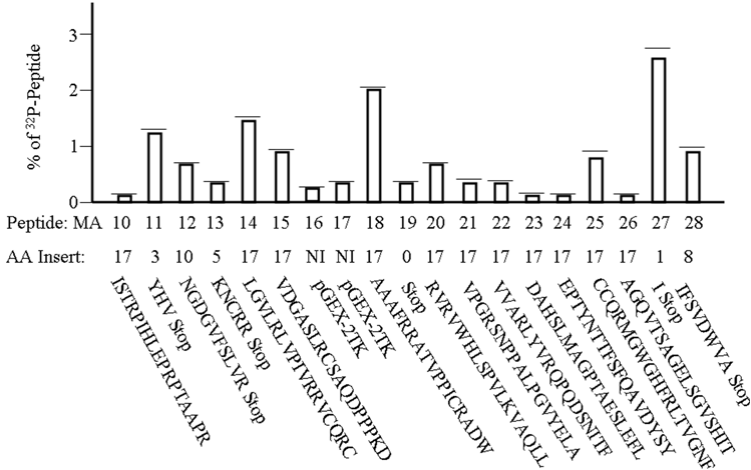
Levels of binding of various ^32^P-labeled peptides to Caco2 cells. Different ^32^P-labeled peptides were incubated for two hours with 10,000 Caco2 cells, washed three times, and incubated in complete medium for 24 hours. The amount of ^32^P radioisotope that remained permanently incorporated into cellular proteins is shown as a percentage of uptake of the amount of peptide added to each cell culture well (mean plus one standard deviation). The number of amino acids present in each insert is shown and ranged from 0 to 17 amino acids. The amino acid sequence of each insert is shown beneath the level of ^32^P incorporation attributed to each insert.

**Figure 2** shows the dramatic variation in levels of permanent ^32^P incorporation into the colon adenocarcinoma line Caco2 after washing and overnight medium incubation. We previously showed that cells successfully binding decapeptides after two hours of incubation released up to 88% of their initially bound ^32^P into media after overnight incubation, but still permanently incorporated high levels of radioisotope into their proteins. The nineteen different peptides in **Figure 2** are designated MA (**M**odified **A**djuvant) 10 through 28. Eleven of these 19 contain complete 17-residue inserts, with MA18 permanently transferring ^32^P to Caco2 cells over 37 times more efficiently than MA26. The most efficient permanent radioisotope incorporation into Caco2 cells occurred after incubation with MA27, which contains only one randomly inserted amino acid upstream of a stop codon. Peptides MA16 and MA17 were encoded by the original recombinant expression vector, leading to low levels of radioisotope incorporation.

### Visualization of ^32^P incorporation by gel autoradiography

**Figure 3:**
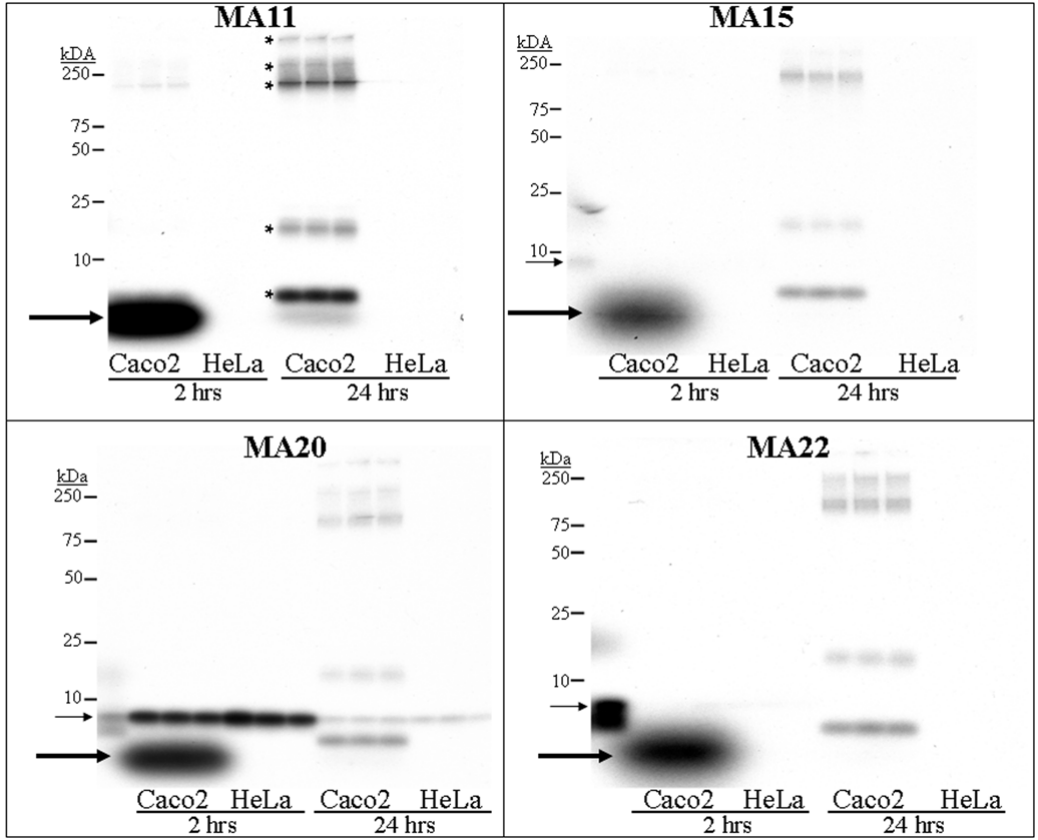
SDS-polyacrylamide gels of ^32^P-peptide binding and radioisotope uptake by Caco2 cells. Four of the MA (Modified Adjuvant) ^32^P-peptides shown in Figure 2 were incubated with triplicate wells of Caco2 or HeLa cells for two hours. After washing, 100 µl of gel loading buffer was added and the contents were run on SDS-polyacrylamide gels (designated as “2 hours”). Identical wells had complete medium added immediately after the washing step and were incubated for an additional 24 hours, and the well contents were then run on gels (designated as “24 hours”). Film was developed after an overnight exposure showing the apparent permanent incorporation of ^32^P into cellular proteins at 24 hours (marked by * in the MA11 panel). Peptide MA11 bound 215 times more avidly to Caco2 cells than to HeLa cells at two hours, and 150 times more avidly at 24 hours. Peptide MA20 bound well to both Caco2 and HeLa cells at two hours, but only Caco2 cells appeared to possess the cellular machinery needed to incorporate ^32^P into cellular proteins. The thin arrow shows the position of the ^32^P-labeled peptide, while the bold arrow shows the position of a relatively low molecular weight labeled intermediate that was not seen in the HeLa cells.

Four peptides showing average levels of radioisotope incorporation were selected for further study; triplicate-well assays of these peptides are displayed in **Figure 3**. Peptide MA11's insert contained three residues upstream of a stop codon, resulting in a peptide only 12 amino acids in length. Despite its relatively short length, this truncated peptide transferred ^32^P to Caco2 cells 215 times more efficiently than to the cervical tumor derived cell line HeLa at two hours. After washing and overnight incubation in medium, radioactivity retained by Caco2 cells was more than 150 times greater than that retained by HeLa cells. As shown in **Figure 3**, most ^32^P bound to Caco2 cells was present in a low-molecular weight (LMW) component (*bold arrow*) at 2 hours, but at 24 hours most of this radioactivity had been incorporated into several different cellular proteins.

Similar results were observed for peptides MA15 and MA22, both of which contained 17-residue inserts for a total length of 34 amino acids, and both of which incorporated 23 times more ^32^P into Caco2 cells than into HeLa cells after overnight incubation (**Figure 3**). Once again, both MA15 and MA22 showed an intensely radioactive LMW band (*bold arrow*) at 2 hours that had almost completely disappeared at 24 hours, with incorporation of the remaining ^32^P into cellular proteins. Originally, we assumed that this LMW band seen at 2 hours (*bold arrow*) represented intact bound ^32^P-labeled peptide. However, the 34 amino acid peptides MA15 and MA22 identified these intact 34-aa peptide precursors as distinct from the intense smaller MW band (*bold arrows*). Thus, we concluded that the smaller band was a rapidly processed small intermediate molecule, which diminished greatly over the ensuring 24 hours during which the ^32^P was being incorporated into the cellular proteins.

Peptide MA20 also contained a 17-residue insert. This peptide was especially noteworthy, since it was the only one tested that was able to bind to a cell line not derived from colon adenocarcinomas and provided key evidence suggesting a possible cellular processing mechanism. As shown in **Figure 3**, MA20 bound at high levels to both Caco2 and to HeLa cells at two hours. However, the LMW band (*bold arrow*) seen with the other three peptides in **Figure 3** was only visible with Caco2 cells, but not with HeLa cells. After washing and overnight incubation, Caco2 cells appeared to have processed the LMW intermediate band (*bold arrow*) into cellular proteins, while HeLa cells apparently lacked the ability to complete this next step (*i.e.*, no radioactive cellular proteins at these MWs were visualized, and all HeLa bound radioactivity was still at the same molecular weight as the originally bound ^32^P-labeled peptide (*thin arrow*)). The two bands (*thin arrows*, first lane of MA20 and MA22 gels) were the result of incubation of ^32^P-labeled peptide in medium containing serum for two hours at 37°C, and demonstrated apparent partial proteolysis of the peptide during that time.

### Only colon adenocarcinoma cells process bound radioactivity into cellular proteins

**Figure 4:**
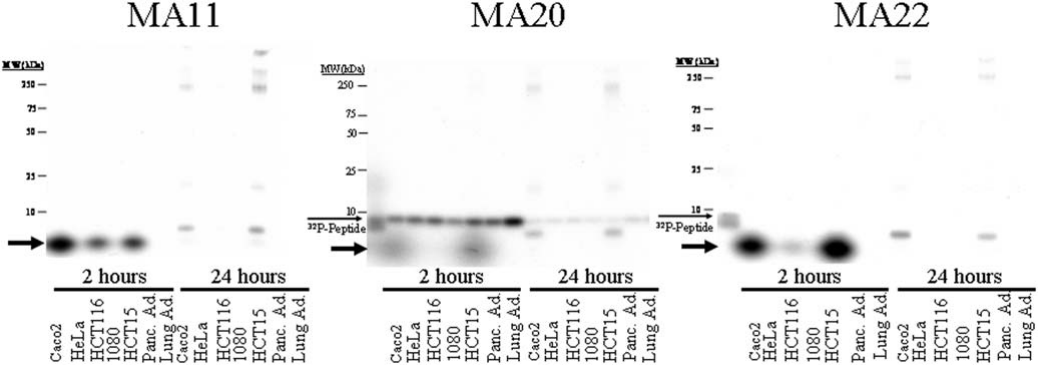
Peptide MA20 binds to multiple cell lines, but ^32^P incorporation is only by colon adenocarcinoma derived lines. The ^32^P-labeled peptides MA11, MA20 and MA22 were incubated with seven different cell lines as described in Figure 3. MA11 and MA22 bound to and had ^32^P radioisotope permanently incorporated by the three colon adenocarcinoma derived cell lines. MA20 significantly bound to all seven cell lines, including one derived from a pancreatic adenocarcinoma and one derived from a lung adenocarcinoma, but had significant levels of ^32^P permanently incorporated into cellular proteins only by the three colon adenocarcinomas. The *thin arrow* shows the position of the ^32^P-labeled peptide, while the *bold arrow* indicates the position of a relatively low molecular weight labeled intermediate that was only seen in colon adenocarcinoma cells.

**Figure 4** displays the results of incubating peptides MA11, MA20 and MA22 with seven different carcinoma cell lines at 2 hours and after overnight incubation. Peptides MA11 and MA22 exhibited strong binding and transfer of ^32^P only to the three colon adenocarcinoma lines (Caco2, HCT116, and HCT15) and not to cervical (HeLa), fibrosarcoma (1080), pancreatic or lung adenocarcinoma cells. MA20, in contrast, bound avidly to all seven cell lines, but its radioactivity was processed into the LMW band (*bold arrow*) and later into cellular proteins only by the three colon adenocarcinoma lines (Caco2, HCT116, and HCT15). HCT116 cells consistently bound, as well as processed into larger-MW bands, radioactivity from all three ^32^P-labeled peptides (MA11, MA20, and MA22) at a much lower rate than Caco2 and HCT15 cells, but incorporation into HCT116 cellular proteins was eventually visible on longer exposures (data not shown).

### Non-phosphorylated peptide competes with ^32^P-labeled peptide for binding to Caco2 cells

**Figure 5:**
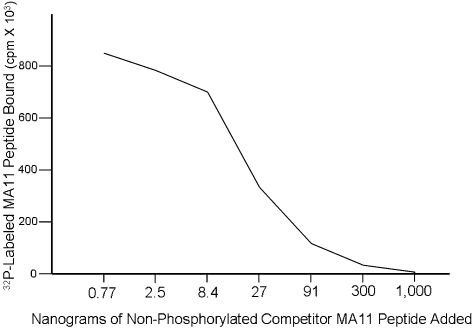
Non-phosphorylated peptide effectively competes with ^32^P-labeled MA11 peptide in binding to Caco2 cells. Into each well containing Caco2 cells was added 0.005 µg ^32^P-labeled MA11 peptide and the indicated quantity of cold, non-phosphorylated MA11. After one hour of incubation, adherent cells were washed and the bound radioactive counts determined.

The 12-aa peptide MA11 was chemically synthesized, labeled with ^32^P, and used in a competitive binding assay with Caco2 cells against varying amounts of cold, non-phosphorylated MA11 peptide. **Figure 5** illustrates that phosphorylation of this peptide was not required to successfully compete for binding to Caco2 cells, and that increasing amounts of cold competitor rapidly inhibited the amount of ^32^P-labeled peptide that bound to cells.

## Discussion

Nineteen different small peptides up to 34 amino acids in length have been recombinantly produced, each containing an insert up to 17 residues long, which can be labeled at a conserved nine amino acid substrate using ^32^P and protein kinase A. These ^32^P-labeled peptides bind with unique affinities to cell lines established from different colon adenocarcinomas and permanently transfer radioisotope to cellular proteins after two hours of incubation. The most efficiently binding peptide results in the permanent uptake of ^32^P by colon cancer cells over 150 times higher than by cell lines derived from other cancers or normal tissues. In addition, one ^32^P-labeled peptide bound to all cell lines tested, but ^32^P was processed and permanently incorporated only by cell lines derived from colon adenocarcinomas, implying that only this type of cancer cell possesses the machinery necessary for this processing step. The nineteen different peptides shown in Figure 2 were selected from an initial screening panel containing only 25 peptides. This surprisingly high rate of obtaining successful peptides enhances the likelihood that this strategy for individualized therapy development will be feasible. Finally, a competitive binding assay using cold and ^32^P-labeled synthetic MA11 peptide demonstrated that non-phosphorylated peptide competes very efficiently for binding to Caco2 cells.

Most currently approved cancer immunotherapeutic regimens use an antibody directed against a known cellular molecule and may also be coupled to a tumor-ablating agent, such as a radioisotope or a toxin [14]–[18]. Only two radioimmunotherapeutic (RIT) treatments are presently FDA-approved; both are directed against non-Hodgkin's lymphoma utilizing ^131^I (Bexxar) or ^90^Y (Zevalin) via the cell-killing activity of emitted electrons. ^32^P radioisotope is a pure beta emitter, and as shown in **Table 1**, it has many properties that compare favorably to ^131^I and ^90^Y, in addition to being readily available and relatively inexpensive. One advantage of using beta particles to kill tumor cells is that their path range of up to 5 mm results in a large number of cells being penetrated by each electron, leading to a cumulative “bystander effect” due to crossfire from neighboring labeled cells.

**Table 1:** Comparison of Properties of Radioactive Beta Emitting Radioisotopes.

Radioisotope	Emits	Maximum beta Energy (MeV)	Range	Half-life (days)
^131^Iodine	Beta	0.6	1.6 mm. (avg.)	8
^90^Yttrium	Beta	2.3	5 mm. (avg.)	2.7
^32^P	Beta	1.7	Up to 5 mm.	14.3

Bexxar (^131^I-anti-CD20) and Zevalin (^90^Y-anti-CD20) are FDA-approved for the treatment of non-Hodgkin's lymphoma.

A very active area of biomedical research focuses on the coupling of radioisotope to peptides, as well as its use in diagnostic and therapeutic applications [19]–[22]. Our proposed application of ^32^P-labeled small peptides in peptide binding therapy suggests a number of advantages over traditional RIT based on monoclonal antibodies. For example, smaller therapeutic molecules are expected to provide better tumor penetration, and the average small peptide molecular weight of less than 4,000 Da is less than 3% of the size of an antibody molecule [23]. Radioactive halogens such as ^131^I can be processed and released prematurely by cells, while the ^32^P delivered by these small peptides is permanently incorporated into cancer cell proteins [24]. A small peptide is less likely to incite the type of host anti-protein response that can develop when using the much larger antibodies, and the absence of an Fc immunoglobulin fragment should result in less nonspecific binding by the liver. The radioisotope ^32^P has a long history of clinical use dating to the early 1930's, while today it is still used to treat polycythemia and essential thrombocythemia [25]. There is a clear need for the development of effective new treatments for colorectal cancer [26]. Our work suggests that an extremely large library of different small peptides, each with unique binding and ^32^P transfer abilities, can be readily produced either chemically or biologically, thus increasing the feasibility of developing individualized treatment regimens for different patients. Cancer has been shown to be a highly heterogeneous disease, thus the development of these unique peptide binding therapies could greatly facilitate individualized patient treatments.

## Materials and Methods

### Production of the recombinant ^32^P-labeled peptides

As described in Figure 1, PCR generated products consisting of 17 random codons flanked by BamHI sites were cloned into the BamHI site of pGEX-2TK (GE Healthcare). After transformation into DH5α bacteria, isolated clones were grown overnight in LB-amp broth, diluted 1/10 in same, grown for two hours, IPTG added to 1 mM, and grown at 37°C for five hours. Ten ml of culture were centrifuged and resuspended in 1 ml of 1×TBS containing 100 µg/ml lysozyme. After two freeze-thaw cycles, the lysate was centrifuged and mixed with 100 µl Sepharose-Glutathione for one hour, washed three times with 1×TBS, and the bound recombinant fusion proteins labeled using ^32^P-γ-ATP and protein kinase A according to the manufacturer's instructions (Sigma, St. Louis, Mo.). The pellet was washed three times with 1×PBS and the labeled peptide was cleaved and released into the supernatant using thrombin (GE Healthcare). For each recombinant peptide produced and assayed, the DNA sequence of the insert in the expression plasmid was determined.

### Assay of the binding of ^32^P-labeled peptides to cell lines

Cell lines were grown in complete medium containing 10% heat inactivated bovine fetal serum. In each well of a 96-well plate, 10,000 cells from various cell lines were grown overnight. Ten µl of the ^32^P-labeled peptide in 1×PBS and 90 µl complete medium were added to each well and incubated at 37°C for two hours. The peptide-medium was removed and one µl added to 100 µl gel loading buffer for scintillation counting for the probe quantitation or run on a 10%–20% polyacrylamide-SDS gel (Biorad). The adherent cells were briefly and gently washed with complete medium three times and some wells were assayed immediately by adding 100 µl of gel loading buffer to each well and run on a gel or counted in a scintillation counter. Other identically treated wells had 200 µl complete medium added and incubated at 37°C for an additional 24 hours. The medium was removed and 100 µl gel loading buffer added and the samples run on a gel or counted as described above.

### Production of synthetic ^32^P-labeled peptide

The 12 amino acid peptide MA11 was chemically synthesized and 0.2 µg was labeled as described above using 300 µCi of ^32^P-γ-ATP and 30 units of protein kinase A. After a five hour labeling reaction, the mixture was microfuged though a Microcon-10 unit to remove the enzyme from subsequent binding assays. For the competitive binding assay, 0.005 µg of ^32^P-labeled peptide MA11 was added to a well containing 10,000 Caco2 cells and a designated quantity of cold, non-phosphorylated MA11. After incubation for one hour, the adherent cells were gently washed and the well contents counted.
